# Analysis of reported cases of sexual violence in Espírito Santo, southeastern Brazil, 2011–2018

**DOI:** 10.1186/s12889-023-15722-8

**Published:** 2023-05-19

**Authors:** Franciéle Marabotti Costa Leite, Beatriz Ferrari, Karina Fardin Fiorotti, Márcia Regina de Oliveira Pedroso, Bruna Venturin, Nicole Letourneau, Fábio Lúcio Tavares

**Affiliations:** 1 Health Sciences Center, Federal University of Espírito Santo, Vitória, ES Brazil; 2 Center for Biological and Health Sciences, University of Western Bahia, Barreiras, BA Brazil; 3 Research Center in Epidemiology, University of Pelotas, Pelotas, RS Brazil; 4 Faculty of Nursing, University of Calgary, Calgary, AB, Canadá Canada

**Keywords:** Sex offenses, Epidemiology, Aggression, Violence against women, Violence

## Abstract

**Background:**

sexual violence includes all sexual acts consummated or attempt to obtain them, unwanted sexual comments and actions that go against the other’s sexuality through coercion, which can be done through the use of physical force, psychological pressure, extortion or threat, this phenomenon appears in all life cycles. Identified the frequency and characteristics of sexual violence against women in a state in the southeastern region of Brazil. from 2011 to 2018.

**Method:**

this is a cross-sectional epidemiological study that evaluated all cases of sexual violence reported in Espírito Santo, present in the Information System of Diseases and Notifications of the Ministry of Health from 2011 to 2018. The analysis was based on performed in Stata 14.1.

**Results:**

the prevalence of notification of sexual violence was 13.2% (CI95%: 12.8–13.5). Most victims were women (PR: 3.38), aged between 0 and 9 years (PR: 19), with a higher prevalence in people without disabilities or disorders (PR: 1.18) and residents of urban/periurban area (PR: 1.15). Men were the most frequent aggressors (PR: 13.79), with the most prevalent cases being reported by people unknown to the victim (PR: 6.01). The occurrence was 78% more reported at home and committed by an aggressor (PR:1.19). Most cases were repeated (PR:1.13).

**Conclusions:**

the notification of sexual violence in Espírito Santo was high and evidenced the vulnerability of some groups, as well as the profile of the perpetrators. It is necessary to work on training professionals in the areas of health and education in relation to the identification of cases of sexual violence, mainly due to the significant involvement of children and adolescents.

## Background

Sexual violence, according to the World Health Organization (WHO), includes all sexual acts consummated or attempt to obtain them, unwanted sexual comments and actions that go against the other’s sexuality through coercion, which can be done through the use of physical force, psychological pressure, extortion or threat [1]. The aggressor can be any individual, regardless of the relationship with the victim, who uses the fragility of the victim to carry out violent acts without its consent. Among the activities, there are rapes by known and/or unknown people, sexual harassment, attempted consummation of the sexual act and others forms [2].

This phenomenon appears in all life cycles. Oliveira and collaborators (2021) highlighted that sexual violence is the main type of injury in children up to 9 years of age in Amazonas-Brazil between 2009 and 2016 [3]. Santos et al. (2019) concluded that students under the age of 13 were victimized more often in the country in 2014 [4]. Both findings demonstrate that the children and youth group in the earlier age groups are more likely to suffer from sexual violence [5]. WHO estimates (2021) point out that one quarter of women aged 15 to 49 years have already been in a relationship where they experienced the aggravation [6]. In the elderly population, sexual violence had a prevalence of 1% when the cases treated in health services were evaluated, between 2011 and 2018, in the state of Espirito Santo, southeastern Brazil [7].

Access to data by the State Health Departments allows the qualification of reports, identification of duplicates and verification of completeness and consistency [8]. Therefore, the systematization of information is more regionalized, recent studies on sexual violence and its characteristics in the municipality of Petrolina and in the states of Santa Catarina, Minas Gerais and Paraná [8–12]. Delziovo and coauthors found a 13% prevalence of sexual violence reports in women aged 10 years or older residing in Santa Catarina, Brazil. This prevalence is higher among adolescents aged 10 to 14 years old, within the home and with a male aggressor [10].

Women and girls who are victims of sexual violence are more likely to suffer from the consequences of sexual and reproductive well-being because they are more frequently affected. Among the main outcomes recommended by the WHO (2018) for this condition in women are unwanted pregnancy, involvement by sexually transmitted infections, unsafe abortion, post-traumatic stress disorder, among others [2]. Meanwhile, children and adolescents who experience sexual violence are subject to psychological, physical, sexual and social impacts [13].

In this context, the National Humanization Policy of the Ministry of Health of Brazil, inaugurated in 2004, places three pillars for the care of victims of violence, which are reception, manifested by professional ethics and trust, accountability, which refers to the commitment to take care of and forward the cases, and the resoluteness, which refers to the effectiveness of solving such demand [14]. The notification of interpersonal and self-inflicted violence is a health surveillance activity and must be carried out in order to have a dimension of the seriousness of the problem, promote public policies in regions and states most affected by the disease, promote comprehensive care for victims and protect and guarantee their rights. Thus, health professionals become important to remove cases of violence from invisibility, preventing their recurrence and providing subsidies so that the system can organize itself in the fight [15].

Given the above, considering the relevance of the theme, its magnitude and impact on health, the following research problem emerged: “what are the characteristics of sexual violence against women in a Brazilian state? In that regard, this study aimed to identify the frequency and characteristics of sexual violence against women in a state in the southeastern region of Brazil. from 2011 to 2018.

## Method

### Study design and location

This is a cross-sectional epidemiological study with the objective of gathering information on cases of sexual violence in the state of Espírito Santo (ES), Brazil, between 2011 and 2018. A state that belongs to the Southeast region of Brazil and whose capital is the city of Vitória. According to the Brazilian Institute of Geography and Statistics, ES has 4,064,052 inhabitants and a population density of 76.25 inhab/km², being the 14th most populous state in Brazil [16].

### Database, sample and outcome variable

The study was carried out with the data entered in the Information System of Diseases and Notification (SINAN) from Ministry of Health and the database was provided by the Health Secretary State of Espírito Santo. The prevalence of cases of sexual violence, which is the outcome variable (yes; no), was evaluated in the population, regardless of age group, in the period from 2011 to 2018. Were included all cases notified and registered in the database, and were excluded duplicated cases (identified by the notification number, victim’s name and name of victim’s mother. This period was adopted, since violence became a problem of compulsory notification in the country in 2011 [17]. The database was submitted to a qualification process and correction of possible errors and inconsistencies, missing values in each variable were disregarded, so the total number of individuals may vary.

### Independent variables and covariates

The independent variables studied will be the characteristics of the victim, the aggressor and the aggression event. Therefore, the characteristics of victims of sexual violence are: gender (male; female); age group (0 to 9 years; 10 to 19 years; 20 to 59 years; 60 years and over); skin color (white; black); presence of disabilities or disorders (no; yes) and the area of residence (urban/periurban; rural). The information of the aggressor evaluated: age group (0 to 19 years; 20–59 years; 60 years or older); sex (male; female; both); relationship with the victim (family member; known; unknown); suspected alcohol use (no; yes). In relation to the occurrence of aggression also were analyzed: the number of people involved (one; two or more); place of occurrence (residence; public highway or others); if it is repeated violence (yes; no) and referral to health services or others (yes; no).

### Statistical analysis

Data were analyzed using the statistical program Stata® version 14.1. The results were expressed as absolute and relative frequency, as well as the 95% confidence intervals (CI95%). For a bivariate analysis, Pearson’s chi-square (x^2^) test was used in the bivariate analysis. In the multivariate analysis, to obtain the association between cases of sexual violence and the independent variables and covariates, the crude and adjusted prevalence ratios (PR) and their CI95% were calculated, according to a Poisson regression model with robust variance. The variables were inserted in the hierarchical model from three levels: in the first, the characteristics of the victim were inserted, second the characteristics of the aggressor and the last variables of aggression event.

### Ethical aspects

This study was approved by the Research Ethics Committee of the Federal University of Espírito Santo identified by registration number 2,819,597.

## Results

Among the reports of interpersonal violence recorded in the state of Espírito Santo in the period studied, sexual violence was in 13.2% (CI95%: 12.8–13.5) of the cases. Table 1 shows that the victims were mainly female (88.2%), aged between 10 and 19 years (42.1%), with black or brown skin color (71.1%), who did not have disability or disorders (90.4%) and resided in the urban/periurban area (92.1%). The aggressor, mostly men (96.1%), aged between 20 and 59 years (74.1%), known (39.2%) of the victim and who had not ingested alcohol at the time of the aggression (64.4%). As for the event of aggression, in most cases, there was the participation of a single aggressor (86.2%), it occurred in the residence (71.3%) and without repetition (52.7%) (Table 2).

**Table 1:** General characteristics of sexual violence cases registered in SINAN from 2011 to 2017 (N = 4,033). Espírito Santo, Brazil, 2011–2018

Victim Characteristics	N(%)	IC 95%
Gender (N = 4,574)		
Male Female	541(11.8) 4,033(88.2)	10.9–12.8 87.2–89.1
Age group (N = 4,574)		
0 to 9 years	1,279(28.0)	26.7–29.3
10 to 19 years	1,924(42.1)	40.6–43.5
20 to 59 years	1,332(29.1)	27.8–30.5
60 years or more	39(0.8)	0.6–1.2
**Skin Color (N = 4,051)**		
White	1,171(28.9)	27.5–30.3
Black	2,880(71.1)	69.7–72.5
**Disabilities or disorders (N = 4,209)**		
No	3,805(90.4)	89.5–91.3
Yes	404(9.6)	8.7–10.5
**Area of residence (N = 4,494)**		
Urban/Periurban	4,138(92.1)	91.3–92.8
Rural	356(7.9)	7.2–8.8
**Characteristics of the agressor**		
**Age group (N = 2,393)**		
0 to19 years	530(22.1)	20.5–23.9
20 to 59 years	1,773(74.1)	72.3–75.8
60 years or more	90(3.8)	3.1–4.6
**Gender (N = 4,310)**		
Male	4,143(96.1)	95.5–96.7
Female	88(2.1)	1.7–2.5
Both	79(1.8)	1.5–2.3
**Relationship with the victim (N = 4,235)**		
Family member	1,536(36.3)	34.8–37.7
Known	1,661(39.2)	37.8–40.7
Unknown	1,038(24.5)	23.2–25.8
**Suspected alcohol use (N = 2,472)**		
No	1,591(64.4)	62.5–66.2
Yes	881(35.6)	33.8–37.6
**Characteristics of occurrence**		
**Number of people involved (N = 4,194)**		
One	3,617(86.2)	85.2–87.3
Two or more	577(13.8)	12.8–14.8
**Place of occurrence (N = 3,986)**		
Residence	2,841(71.3)	69.9–72.7
Public Highway	648(16.3)	15.1–17.4
Others	497(12.4)	11.5–13.5
**Repeated violence (N = 3,670)**		
No	1,935(52.7)	51.1–54.3
Yes	1,735(47.3)	45.7–48.9
**Referral (N = 4,525)**		
No	329(7.3)	6.6–8.1
Yes	4,196(92.7)	91.9–93.5

N: absolute frequency;

%: relative frequency;

CI 95%: confidence interval of 95%

Source: Notifiable Diseases Information System (SINAN), 2011 a 2018

In the bivariate analysis, sexual violence was related to all characteristics of the victim, the aggressor and the event (p < 0.005) (Table 2).

**Table 2:** Bivariate analysis of the distribution of characteristics according to reports of sexual violence in Espírito Santo, Brazil, 2011–2018. (N = 4,033). Espírito Santo, Brazil, 2011–2018

Victim Characteristics	N(%)	CI 95%	p-valor
**Gender**			< 0.001
Male	541(6.1)	5.6–6.6	
Female	4,033(15.6)	15.2–16.1	
**Age group**			< 0.001
0 to 9 years	1,279(41.3)	39.6–43.0	
10 to 19 years	1,924(21.8)	21.0-22.7	
20 to 59 years	1,332(6.4)	6.1–6.7	
60 years or more	39(2.0)	14.7–27.3	
**Skin Color**			0.003
White	1,171(12.6)	11.9–13.3	
Black	2,880(13.8)	13.4–14.3	
**Disabilities or disorders**			< 0.001
No	3,805(15.4)	14.9–15.8	
Yes	404(9.0)	8.2–9.9	
**Area of residence**			< 0.001
Urban/Periurban	4,138(13.5)	13.1–13.8	
Rural	356(10.6)	9.6–11.7	
**Characteristics of the aggressor**			
**Age group**			< 0.001
0 to19 years	530(12.8)	11.8–13.8	
20 to 59 years	1,773(10.1)	9.7–10.6	
60 years or more	90(13.9)	11.4–16.8	
**Gender**			< 0.001
Male	4,143(20.4)	19.9–21.0	
Female	88(0.9)	0.7–1.1	
Both	79(6.3)	5.1–7.8	
**Relationship with the victim**			< 0.001
Family member	1,536(0.9)	10.4–11.5	
Known	1,661(28.9)	27.7–30.1	
Unknown	1,038(29.9)	28.4–31.4	
**Suspected alcohol use**			0.001
No	1,591(11.9)	11.4–12.5	
Yes	881(10.4)	9.8–11.1	
**Characteristics of occurrence**			
**Number of people involved**			0.024
One	3,617(13.4)	13.0-13.8	
Two or more	577(12.1)	11.2–13.1	
**Place of occurrence**			< 0.001
Residence	2,841(13.0)	12.6–13.5	
Public Highway	648(11.9)	11.1–12.8	
Others	497(15.3)	14.1–16.5	
**Repeated violence**			< 0.001
No	1,935(15.7)	15.0-16.3	
Yes	1,735(12.4)	11.9–13.0	
**Referral**			< 0.001
No	329(6.2)	5.6–6.9	
Yes	4,196(15.2)	14.8–15.6	

N: absolute frequency;

%: relative frequency;

CI 95%: confidence interval of 95%

In the multivariate analysis, the majority of victims of sexual violence were women (PR: 3.38; CI95% 3,11 − 3,67), aged between 0 and 9 years (RP: 19.02; CI95% 13,67 − 26,45), with a higher prevalence in people without disabilities or disorders (PR: 1.18; CI95% 1,08 − 1,30) and residents of the urban/periurban area (PR: 1.15). Men were the aggressors (PR: 13.79; CI95% 9,84 − 19,32), with the most prevalent cases being reported from people unknown to the victim (PR: 6.01; CI95% 5,36 − 6,74). The occurrence was 78% more reported in the residence and committed by an aggressor (RP:1.19; CI95% 1,06 − 1,34). Most cases were repeated (RP:1.13) (Table 3).

**Table 3:** Multivariate analysis with the crude and adjusted prevalence ratio of variables associated with cases of sexual violence. Espírito Santo, Brazil, 2011–2018. Espírito Santo, Brazil, 2011–2018

Victim Characteristics	Crude analysis			Adjusted analysis		
	PR	CI 95%	p-value	PR	CI 95%	p-value
**Gender**			< 0.001			< 0.001
Male	Ref.			Ref.		
Female	2.58	2.37–2.82		3.38	3.11–3.67	
**Age group**			< 0.001			< 0.001
0 to 9 years	20.61	15.06–28.20		19.02	13.67–26.45	
10 to 19 years	10.9	7.97–14.91		9.89	7.12–13.75	
20 to 59 years	3.18	2.32–4.36		2.59	1.86–3.61	
60 years or more	Ref.			Ref.		
**Skin Color**			0.003			0.886
White	Ref.			Ref.		
Black	1.1	1.03–1.17		1.01	0.95–1.07	
**Disabilities or disorders**			< 0.001			< 0.001
No	1.70	1.54–1.88		1.18	1.08–1.30	
Yes	Ref.			Ref.		
**Area of residence**			< 0.001			0.005
Urban/Periurban	1.27	1.15–1.41		1.15	1.04–1.27	
Rural	Ref.			Ref.		
**Characteristics of the aggressor**						
**Age group**			< 0.001			0,753
0 to19 years	1.26	1.15–1.38		1.04	0.94–1.16	
20 to 59 years	Ref.			Ref.		
60 years or more	1.37	1.13–1.67		1.02	0.82–1.28	
**Gender**			< 0.001			< 0.001
Male	23.54	19.08–29.03		13.79	9.84–19.32	
Female	Ref.			Ref.		
Both	7.22	5.36–9.73		4.47	2.76–7.21	
**Relationship with the victim**			< 0.001			< 0.001
Family member	Ref.			Ref.		
Known	2.64	2.48–2.81		3.14	2.87–3.45	
Unknown	2.73	2.55–2.93		6.01	5.36–6.74	
**Suspected alcohol use**			0.001			0.002
No	1.14	1.06–1.23		1.13	1.04–1.22	
Yes	Ref.			Ref.		
**Characteristics of occurrence**						
**Number of people involved**			0.024			0.003
One	1.10	1.01–1.19		1.19	1.06–1.34	
Two or more	Ref.			Ref.		
**Place of occurrence**			< 0.001			< 0.001
Residence	1.09	1.01–1.18		1.78	1.60–1.99	
Public Highway	Ref.			Ref.		
Others	1.28	1.15–1.43		1.22	1.07–1.39	
**Repeated violence**			< 0.001			0.001
No	Ref.			Ref.		
Yes	0.79	0.75–0.84		1.13	1.05–1.22	

PR: prevalence ratio;

CI 95%: confidence interval of 95%

Ref.: reference group

## Discussion

The present study points to a significant prevalence of reported cases of sexual violence (P: 13.2%; CI95%: 12.8–13.5). In children and adolescents, as well as in adult women, this type of violence predominated as the second most frequent type [18–19].

As for the characteristics of the victims, it is noted that women were about 3.4 times more victimized compared to men. When evaluating the epidemiological profile of cases admitted to a hospital in Maringá, Paraná, the largest number of female victims was found, corroborating the data of the research in question [20]. Around the world, women are often more victims of sexual violence than men, a result of the interference of cultural and religious factors, social norms and distorted concepts, which make gender relations even more unequal [21].

Violence against women is built on unequal relationships between men and women. It is a brutal expression of machismo. In Brazil, this problem is old, being taken as a system of values that institutes, reinforces and legitimizes the domination of men over women. This inequality is the result of a symbolic violence recognized in the social imaginary, and of a social, cultural and historical construction of being a man and a woman. The understanding of machismo is based, for the vast majority of men and women, on the reference and legitimation to the exercise of weight socially attributed to men in public and private spaces. Still, with regard to male virality, it has as its structuring axis the exercise of an instinctive, active and heteronormative sexuality. Virality concerns a social construction of a hegemonic masculinity, honor. The change in this scenario refers to social changes, values and practices of the social subjects involved [22].

Although in recent decades, Brazil has sought to align itself with the international agendas promoted especially by the United Nations (UN) and, in other multilateral forums with regard to the promotion of gender equality [23], however, the antagonism to the social movements, and the claims for equal rights, practiced by the previous Government, whose presented logic was exclusionary, when addressing issues concerning women, leads to a strengthening of an essentialist perspective, in a very distant way, from political and theoretical constructions of gender concepts, and, is, a complex production of resistance [24]. In this scenario, the gender discussion needs to be rescued and strengthened, which is an indispensable element in the prevention of violence against women [22–24].

Another more vulnerable group that appeared among the notifications was the age group from 0 to 9 years old. In Brazil, 58,037 cases of sexual violence against children were reported between 2011 and 2017, with an increase of 64.6% during the years [25]. This result leads us to reflect on the culture of rape that makes it clear that from childhood women and girls are imposed on a system of submission to men, which ends up supporting violent acts with a character of female victimology [26]. In addition, it is a reflection of intergenerational power relations, which make children vulnerable to meeting the needs and desires of adults [27].

The approach to sexuality and gender in schools is an important tool in the fight against sexual violence against children and adolescents, since it guides and strengthens the role of the school as an institution that supports the right to health, as well as in supporting sexual education [28]. Unfortunately, in recent years, Brazil has faced strong political rebuke regarding the approach to the subject in schools. In 2020, the most recent episode, the Ministry of Women, Family and Human Rights and the Ministry of Health of the Bolsonaro government launched the “Adolescence First, Pregnancy Later” campaign, which, according to experts, goes back to sexual abstinence as a prevention measure, inserting religious and conservative precepts that will not solve the problem [29]. In this sense, sex education currently needs to have its discussion resumed, to be effectively applied in schools, and thus be another tool in the prevention of sexual violence against children and adolescents.

Not having a disability or disorder increased the rate of sexual violence by 18.0%, according to the data analyzed in the survey. As the perpetrators of violence in this type of public are usually family members, caregivers and intimate partners, the possibility of anonymity of the cases is not excluded, which can occur both due to the proximity of the victim to the aggressor, as well as the feeling of shame and dependence on the victim. victim with the same [30].

Male aggressors were the most prevalent, which can be explained by the social roles assigned to men and women in society. Men have always been placed in a position of domination and occupation of positions of power, while women, in a position of submission, fragility and vulnerability. In this context, violence would be a way of reaffirming male domination [31]. And this fact occurs in all cycles of a woman’s life, from childhood to adulthood [10].

The probability of the aggressor being unknown to the victim increased the prevalence of notification of sexual violence, which is similar to what was said by Santarem et al. (2020) when evaluating the cases registered at the Gynecological Emergency Unit of the Hospital in southern Brazil [32]. This can be explained by the fact that aggressions carried out by people known to the victim tend not to be notified due to their proximity to the aggressor [13]. Being assaulted by a single aggressor was also more prevalent, which is in agreement with the data presented by Teixeira et al. (2019) when citing that 85.9% of victims of sexual violence between 2006 and 2017 were assaulted by a single person [32].

It is important to note that most cases of sexual violence occurred at home, which was also identified in another study [10]. This aspect retains importance when considering that underage victims are usually assaulted by people from their own family nucleus. Research carried out in Pernambuco, which analyzed reports of sexual violence against children and adolescents, indicated that the father and stepfather are 8 times more likely to be the aggressor in these cases [5]. This indicates that the home loses its protective function, since often the aggressors are family members who live in the same home as the victim and who take advantage of the fact to maintain their condition in anonymity [33].

The notification of sexual violence was more prevalent in the urban/periurban area, which is similar to data from a study carried out with victims of sexual violence in the Amazon region [20, 34]. It is believed that this fact is due to the lack of resources in regions further away from the centers, which contributes to underreporting in these places [34].

Recidivism of sexual victimization was identified in the study. This result points to the importance of the health professional in the early detection of these situations, which permeates the orientation of the victim about his role in the end of the violence [35]. In a study carried out in Santa Catarina, recidivism increased by 1.69 times the rate of involvement during pregnancy, one of the most frequent outcomes in sexual violence. Living with the potential aggressor, often in their own home, favors not looking for the health service and the continuity of the cycle of violence [36].

Among the limitations of the study is the representativeness and underreporting of cases of sexual violence, since when working with data from information systems such as Sinan, the reported cases are those seen and identified in health services. Many cases of sexual violence occur and go unreported. It should also be noted, as one of the limitations of the study, the inherent difficulties in using secondary data, such as its accuracy and completeness. In this sense, the constant improvement of the surveillance process and the permanent training of health professionals are essential for the adequate characterization of cases and the correct completion of the notification form.

The science in Brazil has been profoundly marked by advances and setbacks over the decades, so that factors such as structural conditions of research work, regional differences in a country of continental dimensions, research funding and conflicts between public and private institutions are great challenges for researchers [37].

Epidemiological studies, especially those that, like the present work, address the issue of sexual violence, a phenomenon that is increasingly evident in contemporary societies, have proven to be fundamental insofar as they allow the elucidation of the grievance, contribute to the articulation of necessary actions in the tackling the problem, as well as advancing population data science and science as a whole.

## Conclusion

It was found that the victims were predominantly women, aged between 0 and 9 years, with a higher prevalence in people without disabilities or disorders and who lived in the urban/periurban area. Men were the main aggressors, being mostly unknown to the victim and the occurrence was more reported in the residence and with the presence of an aggressor. Most cases were repeated.

The significant involvement of children and adolescents is worrying, given the physical, emotional and psychological consequences that the various types of sexual violence can cause the victim throughout their lives. Despite the existence of several public policies to combat violence in Brazil, such as the Child and Adolescent Statute, there are still difficulties in implementing these at local levels and this is one of the great factors that also contribute to the continuity of this problem even today. In addition, the lack of articulation between the different government sectors (education, health, social assistance, and public security) makes it difficult to network and monitor victims.

It is necessary to work on training professionals in the areas of health and education regarding the identification of cases of sexual violence, as a way of combating underreporting, which is one of the factors that strengthens recidivism, also reported in the present study.

## Data Availability

Our data is not publicly available because contains identification of participants. Data without identification can be accessed from the Ministry of Health website. The datasets used and/or analyzed during the current study are available from the corresponding author on reasonable request.
